# Regulation of Bone Morphogenetic Protein 9 (BMP9) by Redox-dependent Proteolysis*

**DOI:** 10.1074/jbc.M114.579771

**Published:** 2014-09-18

**Authors:** Zhenquan Wei, Richard M. Salmon, Paul D. Upton, Nicholas W. Morrell, Wei Li

**Affiliations:** From the Department of Medicine, University of Cambridge, School of Clinical Medicine, Box 157, Addenbrooke's Hospital, Hills Road, Cambridge CB2 0QQ, United Kingdom

**Keywords:** Bone Morphogenetic Protein (BMP), Cell Signaling, Crystal Structure, Disulfide, Endothelial Cell, Redox Regulation

## Abstract

BMP9, a member of the TGFβ superfamily, is a homodimer that forms a signaling complex with two type I and two type II receptors. Signaling through high-affinity activin receptor-like kinase 1 (ALK1) in endothelial cells, circulating BMP9 acts as a vascular quiescence factor, maintaining endothelial homeostasis. BMP9 is also the most potent BMP for inducing osteogenic signaling in mesenchymal stem cells *in vitro* and promoting bone formation *in vivo*. This activity requires ALK1, the lower affinity type I receptor ALK2, and higher concentrations of BMP9. In adults, BMP9 is constitutively expressed in hepatocytes and secreted into the circulation. Optimum concentrations of BMP9 are essential to maintain the highly specific endothelial-protective function. Factors regulating BMP9 stability and activity remain unknown. Here, we showed by chromatography and a 1.9 Å crystal structure that stable BMP9 dimers could form either with (D-form) or without (M-form) an intermolecular disulfide bond. Although both forms of BMP9 were capable of binding to the prodomain and ALK1, the M-form demonstrated less sustained induction of Smad1/5/8 phosphorylation. The two forms could be converted into each other by changing the redox potential, and this redox switch caused a major alteration in BMP9 stability. The M-form displayed greater susceptibility to redox-dependent cleavage by proteases present in serum. This study provides a mechanism for the regulation of circulating BMP9 concentrations and may provide new rationales for approaches to modify BMP9 levels for therapeutic purposes.

## Teaser

**Background:** Bone morphogenetic protein 9 (BMP9) circulates at low concentrations maintaining endothelial integrity. BMP9 has potent bone-forming activity at high concentrations.

**Results:** The intermolecular disulfide bond in BMP9 is regulated by redox potential; the loss of which renders BMP9 susceptible to degradation by proteases present in serum.

**Conclusion:** BMP9 is regulated by redox-dependent proteolysis.

**Significance:** Optimum circulating BMP9 levels are essential for endothelium-specific activity.

## Introduction

BMP9 is a circulating vascular quiescence factor (1), one of only two BMP^5^ ligands that specifically activate the endothelial ALK1/bone morphogenetic protein receptor type II (BMPR-II) pathway (2). ALK1 is an endothelial-specific type I receptor (3), and BMPR-II is a type II receptor for the large family of BMP ligands (4). ALK1 and BMPR-II play essential roles in early developmental processes. Homozygous knock-out ALK1 or BMPR-II in mice are embryonic lethal due to defects in early heart and vessel development (5, 6). Human mutations in ALK1 lead to type II hereditary hemorrhagic telangiectasia (3), a vascular dysplasia of multiple telangiectasias and arteriovenous malformations in internal organs typically affecting the lung, brain, gastrointestinal tract, and liver, which can cause life-threatening hemorrhage (7). Mutations in BMP9 have been identified in patients with a vascular disorder phenotypically overlapping with hereditary hemorrhagic telangiectasia (8). Human mutations in BMPR-II are the commonest genetic cause of pulmonary arterial hypertension (PAH), characterized by increased pressure in the pulmonary circulation due to narrowing of the lung blood vessels, which leads to right ventricular hypertrophy and death within a few years of diagnosis (9, 10). ALK1 mutations were found in occasional PAH patients (11, 12), and ALK1^+/−^ mice spontaneously develop PAH (13).

Endothelial dysfunction, characterized by endothelial cell apoptosis and increased endothelium permeability, is a recognized trigger for PAH, and the ALK1/BMPR-II pathway plays an essential role in maintaining the endothelium integrity (14, 15). Loss of BMPR-II predisposes human pulmonary artery endothelial cells (hPAECs) to apoptosis and BMPs can inhibit hPAEC apoptosis induced by serum starvation (16). BMPR-II loss leads to the increased permeability of the hPAEC monolayer, reduced endothelial barrier function (14), and PAH (15). Moreover, targeted gene delivery of BMPR-II to the pulmonary endothelium attenuates PAH in rodent models (17).

The endothelial ALK1/BMPR-II pathway is constitutively activated by circulating BMP9, the only confirmed BMP that circulates at active concentrations (18, 19). BMP9 is produced by hepatocytes as the prepro-form and is processed by proprotein convertase in the *trans*-Golgi network into prodomain and mature BMP9 ligand (20). The prodomain of BMP9 forms a complex with mature BMP9 in the circulation but does not affect BMP9 signaling activity (18). BMPR-II on the surface of hPAECs undergoes rapid turnover (21), and BMP9 induces BMPR-II expression in endothelial cells in an ALK1-dependent manner (22).

In addition to its role in the vascular endothelium, BMP9 signaling activity has also been demonstrated in mesenchymal stem cells and C2C12 myoblasts. Among 14 BMPs tested, BMP9 has the highest osteogenic signaling activity *in vitro* and potently induces bone formation *in vivo* (23, 24). No defects in bone or cartilage have been reported in the BMP9 knock-out mice (25, 26), and the role of BMP9-induced osteogenic activity in human physiology is not fully understood, but such potent osteogenic potential of BMP9 raises the question why circulating BMP9 does not show osteogenic activity in blood vessels.

One possible reason for this is the concentration of BMP9. Circulating levels of BMP9 are between 2–10 ng/ml measured by activity (18, 19) and ∼300 pg/ml by ELISA (27). BMP9 signaling in endothelial cells is mediated by the high-affinity receptor, ALK1 (28), whereas BMP9 osteogenic signaling activity requires both ALK1 and the low-affinity receptor, ALK2 (29). Whereas the EC_50_ of BMPs activating ALK2, ALK3, and ALK6 is in the range of 50 ng/ml, BMP9 is particularly potent in activating ALK1, with an EC_50_ of 50 pg/ml (18). Whereas other unknown factors may contribute to the non-osteogenic, endothelial-specific ALK1-mediated signaling by BMP9, optimum concentrations of circulating BMP9 maybe an important factor essential for maintaining endothelial specificity.

BMP9 is constitutively produced by the liver and secreted into the circulation. We hypothesized that there may be mechanisms in place to regulate circulating BMP9 at the optimum levels and activities for ALK1-specific signaling. We addressed this question by characterizing recombinant BMP9 produced in mammalian cells and purified under native, non-denaturing conditions. We demonstrated that BMP9 stability and activity was regulated by redox potential as well as proteolysis. The redox-dependent cleavage of the non-covalently linked BMP9 dimer would provide a controlled natural degradation pathway for BMP9. Such redox-dependent cleavage would suggest that although there is a constant degradation of the BMP9 from the circulation, a fraction of BMP9 (covalently linked BMP9 dimer) remains stable and resistant to proteolysis, ensuring the constitutive activation of the endothelial ALK1/BMPRII pathway to maintain the homeostasis of the vascular endothelium.

## EXPERIMENTAL PROCEDURES

### 

#### 

##### Materials

Anti-BMP9 antibody (MAB3209 and AF3209), anti-BMP9 prodomain antibody (AF3879), and control BMP9 were purchased from R&D Systems, Inc. Anti-His tag antibody (37–2900) was purchased from Invitrogen. Prodomain-bound BMP9 (pBMP9) and BMP6 were kind gifts from Pfizer. Anti-phospho-Smad1/5/8 antibody was purchased from Cell Signaling Technology. HiTrap nickel-nitrilotriacetic acid, HiTrap Q FF, and Superdex 10/30 columns were purchased from GE Healthcare. hPAECs and endothelial growth medium (EGM-2) were purchased from Lonza, UK. All other tissue culture media were purchased from Invitrogen. Crystallization reagents were purchased from Hampton Research, Inc. All plasmid purification kits were purchased from Qiagen.

##### BMP9 Expression and Purification

Human full-length pro-BMP9 cDNA was cloned into pCEP4 between the HindIII and XhoI sites and verified by DNA sequencing. To facilitate the purification of mature BMP9, a His_6_ tag was introduced immediately after the putative furin cleavage site to generate pro-HBMP9. Plasmids containing pro-HBMP9 were transfected into HEK-EBNA cells using polyethylenimine in DMEM medium containing 5% FBS. Cells were changed into CDCHO expression medium without serum the following day, and conditioned media were harvested after 3–4 days. To purify HBMP9, 5 liters of conditioned media were concentrated using a Vivaflow 200 concentrator (Sartorius AG) to 200 ml and dialyzed against 4 liters of 20 mm Tris·HCl, pH7.4, 250 mm NaCl at 4 °C overnight. Samples were loaded onto a 5-ml nickel-nitrilotriacetic acid column in 1× binding buffer (5 mm Tris·HCl, pH 7.4, 500 mm NaCl, 5 mm imidazole). After extensive washing with 1× binding buffer, bound fractions were eluted with an imidazole gradient (5–250 mm) over 10 column volumes. Fractions were analyzed by non-reducing SDS-PAGE, and those containing a mixture of partially processed pro-HBMP9, prodomain, and mature HBMP9 were pooled and dialyzed against 20 mm Tris·HCl, pH 7.4, and loaded onto a 1-ml Q Sepharose column. HBMP9 eluted in the unbound fraction at >95% purity and was used in the characterization and crystallization assays.

##### BMP Response Element Luciferase Assays in C2C12 Cells

C2C12 cells were seeded at 4 × 10^4^ cells/well in 24-well plates. The next day, cells were washed once and incubated in OptiMEM I for 2 h. All wells were then co-transfected with plasmids containing BMP response element luciferase (courtesy of Professor P. ten Dijke, Leiden University Medical Center, Leiden, Netherlands) and *Renilla* luciferase. In the wells for testing BMP9 binding to ALK1, pcDNA3-hALK1 (courtesy of Professor R. C. Trembath, King's College London, London, UK) was also added. After 4 h, the transfection mixtures were removed, and DMEM containing 10% FBS and antibiotic-antimycotic (Invitrogen) added for 24 h. Cells were washed twice with serum-free DMEM containing antibiotic, incubated in serum-free DMEM for 10 h and then treated for 18 h with dilutions of recombinant BMP9 proteins. At the end of the treatment, cells were lysed and assayed for firefly and *Renilla* luciferase activities using the Dual-Glo® luciferase assay kit (Promega) according to the manufacturer's instructions. Firefly luciferase activities were normalized to the *Renilla* control.

##### BMP9 Crystallization and Structure Determination

Crystallization trials were set up with a complex containing HBMP9 and ALK1 extracellular domain (ECD) (1:1 ratio, 4 mg/ml in 20 mm Tris·HCl, pH 7.4, 50 mm NaCl), and diffraction quality crystals were obtained in 0.12 m Mg(NO_3_)_2_, 12% PEG3350 over 3 days. Analysis of the washed crystals by non-reducing SDS-PAGE revealed that the crystals contained equal amounts of D- and M-forms of BMP9, but not ALK1 ECD. Crystals were cryo-protected in 0.12 m Mg(NO_3_)_2_, 22% PEG3350, 20% glycerol, and data were collected at 100 K at Diamond Light Source I04 from a single crystal. Data were processed using Mosflm, Scala, and Truncate (30) to 1.90 Å, and the structure was solved by molecular replacement using 1ZKZ as search model (31, 32). Model building was carried out using Coot (33) and refinement with REFMAC5 (34) and Phenix refine (35). Figures were produced using Coot and PyMOL (The PyMOL Molecular Graphics System, version 1.2r3pre, Schrödinger, LLC.). Crystallographic data and refinement statistics are given in Table 1, and the coordinate files were deposited in the Protein Data Bank under the accession code 4MPL.

##### BMP9 Signaling in hPAEC

HBMP9 C73S and control HBMP9 were expressed in HEK-EBNA cells. To evaluate the concentrations of BMP9 in the conditioned medium, we used Western blot of the conditioned media against an anti-BMP9 prodomain antibody. This is because BMP9 C73S cannot be detected with commercially available anti-BMP9 antibodies, and the BMP9 prodomain is produced in the same peptide chain and processed in a 1:1 ratio with mature BMP9 upon secretion. The ratio of prodomain of C73S to wild type in the transfected medium should be identical to the ratio of mature ligands. The concentration of wild type BMP9 was determined by ELISA using R&D BMP9 as standards and the concentration of the C73S mutant was calculated from the ratio obtained from the prodomain Western blot. For signaling assays, hPAEC were seeded in 60-mm dishes at 3 × 10^5^ cells/dish and cultured for 24 h in EGM-2 medium with 10% FBS. Cells were quiesced in EGM-2 medium with 0.1% FBS for 16 h before treatment with BMP9. At the designated time points, the medium was aspirated, and dishes were placed on dry ice to stop the signaling reaction before lysis buffer (125 mm Tris·HCl, pH 6.8, 2% SDS, and 10% glycerol) was added. The protein concentration in the total cell lysate was determined using DC^TM^ protein assay (Bio-Rad), and 25–35 μg of total cell protein was used for immunoblotting. BMP9 signaling was monitored by the phosphorylation of Smad1/5/8 detected by anti-pSmad1/5/8 antibody. α-Tubulin was used as a loading control.

##### BMP9 Redox Assay and Redox-dependent Cleavage

BMP9 redox assays were carried out by redox titration with glutathione (GSH)/glutathione disulfides (GSSG) adapted from Zhou *et al.* (36). HBMP9 or pBMP9 were incubated with PBS or PBS containing 0.1 mm GSSG with 0 to 20 mm GSH at room temperature overnight in the presence of protease inhibitor (Complete-EDTA, Roche Diagnostics). Samples were fractionated by 12% SDS-PAGE under non-reducing conditions and stained with Coomassie Blue (Simply Blue, Invitrogen).

For redox-dependent proteolysis, HBMP9 or pBMP9 were treated as above, but in the absence of protease inhibitor. The following day, trypsin was added at the indicated concentrations (w/w), and samples were incubated for 3 h for HBMP9 or overnight for pBMP9. All samples were fractionated on a 12% non-reducing SDS-PAGE and stained with Coomassie Blue to detect the cleavage products.

## RESULTS

### 

#### 

##### Expression and Purification of Human BMP9 from Mammalian Cells

**FIGURE 1.**
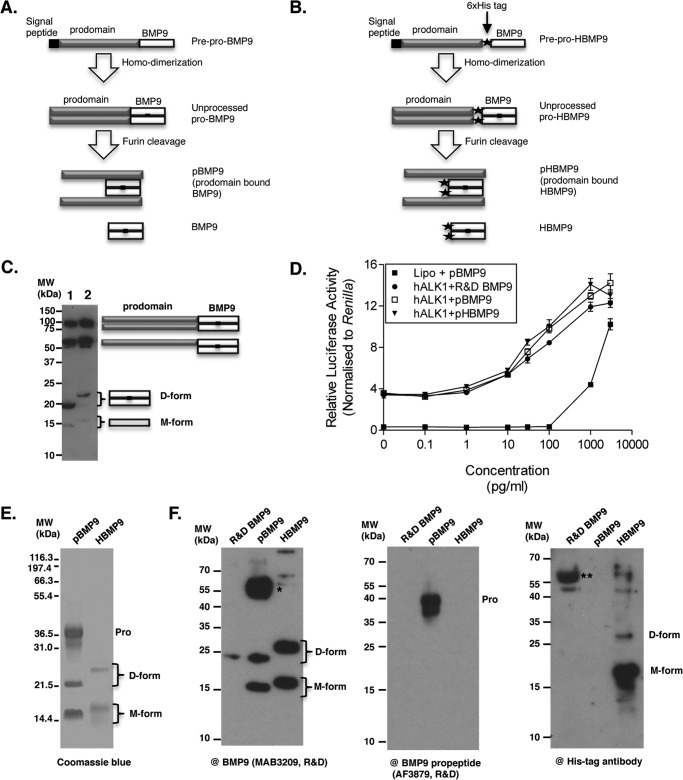
**Expression, activity, and purification of recombinant BMP9.** *A*, schematic diagram of BMP9 production and processing. *B*, schematic diagram of the generation of HBMP9. A His tag (*filled star*) is introduced at the beginning of the mature BMP9. *C*, conditioned medium from HEK-EBNA cells transfected with prepro-BMP9 (*lane 1*) or prepro-HBMP9 (*lane 2*) were separated on a non-reducing SDS-PAGE and probed with anti-BMP9 antibody (MAB3209). The diagram on the *right* shows the schematic drawing of the BMP9 molecules. *D* denotes dimer on non-reducing SDS-PAGE, and *M* denotes monomer on SDS-PAGE. D- and M-forms migrate slower in the HBMP9 due to the addition of the His_6_ tag at the N terminus of the mature ligand as depicted in *B. D*, HEK cell-produced BMP9 and HBMP9 have comparable activity with BMP9 from R&D Systems. Conditioned media containing pBMP9 (because it is very likely to be present as a prodomain bound complex in the conditioned media) or pHBMP9 were quantified by ELISA using R&D BMP9 as a standard and subjected to signaling assay using C2C12 cells transfected with ALK1. Plasmid containing *Renilla* was co-transfected with reporter plasmid containing BMP response element-luciferase, and the luciferase activity induced by BMP9 signaling was read using the Promega Dual-Luciferase system. *E*, purified pBMP9 and HBMP9 were fractionated on a 12% non-reducing SDS-PAGE and stained by Coomassie Blue. *F*, identical samples of R&D Systems BMP9, pBMP9, and HBMP9 were run in parallel on three 12% non-reducing SDS-PAGE, then blotted separately against the following: anti-BMP9 antibody (*left*), anti-BMP9 prodomain antibody (*middle*), and anti-His tag antibody (*right*). A *single asterisk* indicates a minor band that could not be seen on the SDS-PAGE in *E* but reacted very strongly with anti-BMP9 antibody. This may be a species of partially processed BMP9. *Double asterisks* indicate nonspecific carrier protein from R&D Systems BMP9. *Pro*, prodomain.

BMP9 is synthesized as the prepro-form and processed into the mature ligand and the prodomain during secretion (Fig. 1*A*). To ensure the proper folding and processing of BMP9, we used a mammalian transient transfection system to overexpress human BMP9. Full-length pro-BMP9 and pro-HBMP9 (Fig. 1*B*) were overexpressed in HEK-EBNA cells, and transfection supernatants were blotted against anti-BMP9 antibody (Fig. 1*C*). Similar to the pulse-chase experiment from CHO cells transfected with pro-BMP7 (37), both dimeric (D-form) and monomeric (M-form) forms of BMP9, together with the partially processed pro-BMP9, could be readily detected (Fig. 1*C*). Activity assays using BMP response element luciferase-transfected C2C12 cells and BMP9 transfection media showed that pHBMP9 has the same signaling activity as pBMP9 and BMP9 from R&D Systems (Fig. 1*D*).

The mature ligand HBMP9 was purified to >95% purity and both D- and M-forms were co-purified (Fig. 1*E*). We also obtained pBMP9, a purified protein complex of prodomain bound human BMP9 (a kind gift from Pfizer), representing the circulating form of BMP9 in human (Fig. 1*E*). The identities of the bands of pBMP9 and HBMP9 in Fig. 1*E* were confirmed by Western blot using antibodies against BMP9, prodomain, and His tag, respectively (Fig. 1*F*). Both forms of BMP9 and HBMP9 reacted with anti-BMP9 antibody, and only prodomain reacted with the anti-prodomain antibody. Anti-His antibody reacted with both d- and M-forms of HBMP9, but much more potent in detecting M-form BMP9.

Although pBMP9 was generated from a totally different source and by different purification methods, a similar ratio of D- and M-forms of BMP9 was present in the pBMP9 as in HBMP9. Because the presence of stable BMP9 in the absence of an intermolecular disulfide bond and its co-existence with the disulfide-linked form has not been reported for the TGFβ superfamily ligands, we investigated whether the disulfide bond plays a role in regulating BMP9 activity and stability. In the following sections, pBMP9 represented the circulating form and HBMP9 represented mature BMP9, akin to the commercial BMP9 (R&D Systems) that has been used in most of the literature to date.

##### The M-form BMP9 Is a Non-covalently Linked Dimer

**FIGURE 2.**
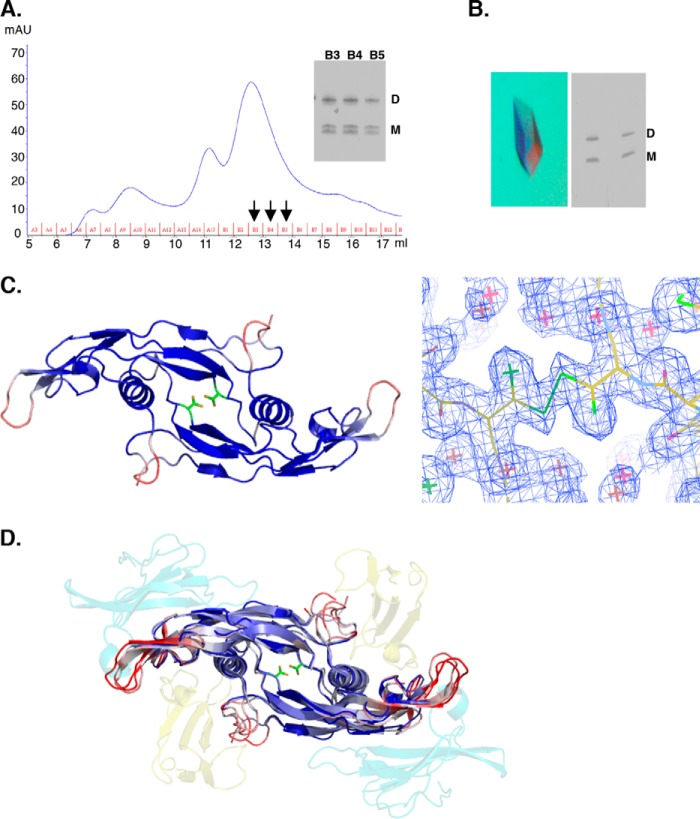
**M-form BMP9 is a non-covalently linked dimer.** *A*, HBMP9 was loaded onto a Superdex 75 10/30 gel filtration column pre-equilibrated in 50 mm Tris·HCl, pH 7.4, containing 150 mm NaCl. Non-reducing SDS-PAGE of the peak fractions (B3 to B5) revealed the D- and M-forms of HBMP9 co-elute under the same peak. The doublet in the M-form on SDS-PAGE was probably due to a partial reduction of intramolecular disulfide bonds. *B*, a representative of the HBMP9 crystal (*left*) and two examples of washed single crystals ran on a non-reducing SDS-PAGE (*right*) demonstrated that each single crystal contains a mixture of D- and M-forms of HBMP9. *C*, crystal structure of HBMP9 (*left*), colored according to the B-factors (spectrum, *blue* to *white* to *red*, from 20 to 100) with Cys-73 in two conformations shown in *sticks*. Electron density (2*F_o_* − *F_c_* map at 1σ) clearly shows two conformations of Cys-73 (*right*). *D*, HBMP9 was overlaid with the published BMP9 structures (Protein Data Bank codes 1ZKZ and 4FAO, all colored as described in *C*). In the semi-transparent schematic, type I receptor ALK1 is shown in *yellow*, and activin receptor type 2B is shown in *cyan* as in BMP9·ALK1·activin receptor type 2B complex (Protein Data Bank code 4FAO).

Forming a stable dimer is essential for BMP signaling activity because the minimum signaling unit for BMP comprises one BMP dimer, two copies of the type II receptor, and one copy of the type I receptor (38, 39). To determine whether M-form BMP9 is a monomer or dimer in solution, semi-purified HBMP9 was passed through a gel filtration column. The D- and M-forms of BMP9 were co-eluted under a single peak, which was confirmed by analyzing the fractions (B3 to B5) on a non-reducing SDS-PAGE (Fig. 2*A*). To confirm the presence of the intermolecular disulfide bond in the D-form and to further delineate any local conformational differences between the two forms of BMP9, crystallization trials were set up, and diffraction quality crystals were grown within a week. A single crystal contained both D- and M-forms of HBMP9 (Fig. 2*B*). Because two previously published BMP9 structures were either in the M-form or the D-form and crystallized under different protein-protein interaction contexts (32, 40), our crystal is unique in addressing the local differences between D- and M-forms of BMP9 under identical conditions. We solved the structure to 1.9 Å with a space group *I*4_1_22 (Table 1). Similar to the published BMP9 structure (Protein Data Bank code 1ZKZ), there was only one HBMP9 monomer in an asymmetric unit, forming a canonical BMP dimer with the symmetry-related molecule. This is the highest resolution BMP9 structure reported so far, showing an overall well ordered molecule with an average B factor of 32.6. Indeed, Cys-73, the critical amino acid involved in disulfide bond formation, was in two conformations as demonstrated by clear electron density (Fig. 2*C*), with an intermolecular disulfide bond formed between the two monomers in one of the conformations. Interestingly, no other major difference could be observed for the remaining residues between the D- and M-forms. Overlaying the HBMP9 structure with the previously published BMP9 structures revealed that the core regions of BMP9 were almost identical. Loops contacting the type I and type II receptors had diverged conformations. These are also the regions with the highest B factors in all BMP9 structures, indicating an intrinsically higher degree of flexibility at these regions (Fig. 2*D*). This structure confirms that stable BMP9 dimers can form with or without an intermolecular disulfide bond.

**TABLE 1:** **Crystals, data processing, refinement, and models**

Crystals	HBMP9 (PDB code 4MPL)*^a^*
Space group	*I*4_1_22
Cell dimensions	*a* = *b* = 71.27, and *c* = 145.89 Å, α = β = γ = 90°
Solvent content (%)	65.66

**Data processing statistics**	
Wavelength (Å)	0.9795
Resolution (Å)	40.17-1.90 (2.00-1.90)
Total reflections	117,427
Unique reflections	15,096
Mn (*I*/sd)	15.6 (2.7)
Completeness (%)	99.3 (98.6)
Multiplicity	7.8 (8.0)
*R*_pim_	0.029 (0.279)

**Model**	
No. of protein atoms	930
No. of water molecules	95
Average B-factor (Å^2^)	32.6

**Refinement statistics**	
Reflections in working/free set	14,327/769
*R*_factor_/*R*_free_	19.8/22.6
r.m.s.d. of bonds (Å)/angles from ideality	0.008/1.198°
Ramachandran plot*^b^*	
Favored (%)	94.44
Outlier (%)	0

*^a^* PDB, Protein Data Bank; r.m.s.d., root mean square deviation.

*^b^* Calculated using Molprobity (52).

##### Both D- and M-forms of BMP9 Can Bind to ALK1 and Prodomain

**FIGURE 3.**
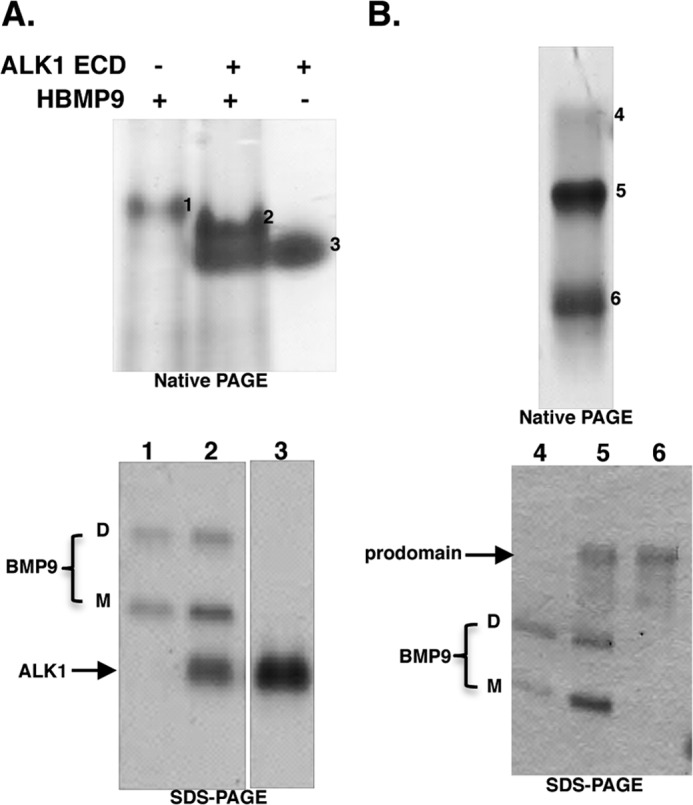
**M-form BMP9 can bind to ALK1 and prodomain as the D-form.** *A*, M- and D- forms of HBMP9 co-migrate on native PAGE, and both can form complexes with ALK1 ECD. *B*, M- and D-forms of BMP9 co-migrate, both as free form and as prodomain-bound form. In *A* and *B*, *bands 1* to *6* from native PAGE were cut out, boiled in 2× SDS-loading buffer for 20 min before loading onto a non-reducing SDS-PAGE to confirm the identities. For SDS-PAGE in *A*, two parts of the same gel are shown.

We next investigated whether the M-form BMP9 maintained protein-protein interactions known for the D-form. Because BMP9 binds to ALK1 ECD with high affinity and circulates as a complex with its prodomain, we examined whether the M-form could form a complex with ALK1 ECD and BMP9 prodomain using native PAGE followed by SDS-PAGE (Fig. 3, *A* and *B*). HBMP9 migrates as a single band on a native PAGE, containing both D- and M-forms (Fig. 3*A*, *band 1*). In the presence of excess ALK1 ECD (Fig. 3*A*, *band 3*), both D- and M-forms of BMP9 formed complexes with ALK1 ECD and shifted to a new band (Fig. 3*A*, *band 2*). To investigate the prodomain-BMP9 interaction, pBMP9 was run on a native PAGE. It fractionated into three bands on the native PAGE. SDS-PAGE analysis of bands 4–6 revealed that both D- and M-forms of BMP9 migrate as a single entity, either as free form (*band 4*) or in the prodomain bound complex (*band 5*). These data agree with the crystal structure that in solution, M-form was indistinguishable from the disulfide-linked D-form. M-form co-migrated with the D-form on the native PAGE, both in free form, and in complexes with ALK1 ECD or prodomain, indicating M-form BMP9 can form complexes with ALK1 ECD and the prodomain as the D-form.

##### The Intermolecular Disulfide Bond Is Not Required for BMP9 Signaling Activity

**FIGURE 4.**
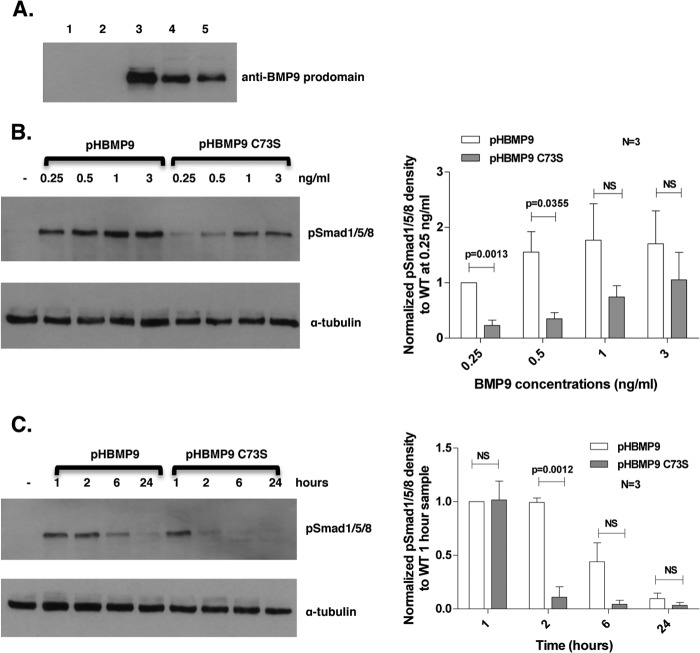
**Intermolecular disulfide bond is not required for BMP9 signaling activity.** *A*, conditioned media (6 μl) from HEK-EBNA cells transfected with transfection reagent alone (*lane 1*), empty vector (*lane 2*), pro-BMP9 (*lane 3*), pro-HBMP9 (*lane 4*), or pro-HBMP9 C73S (*lane 5*) were fractionated on a 12% SDS-PAGE and probed with anti-BMP9 prodomain antibody. The intensities of bands were quantified using ImageJ, and the ratio of HBMP9 C73S to HBMP9 obtained. HBMP9 C73S concentration in the conditioned medium was normalized to HBMP9 using the above ratio. *B*, *left*: hPAECs were serum-restricted in EGM-2, 0.1% FBS overnight, and stimulated with HBMP9 or HBMP9 C73S (both 0.25–3 ng/ml) for 1 h. Cells were harvested, and total cell protein was immunoblotted with anti-pSmad1/5/8 antibody. Band intensities of pSmad1/5/8 blots were analyzed using ImageJ, corrected by ratios obtained from the α-tubulin blot, and normalized to a 0.25 ng/ml wild type sample. Data of the mean ± S.E. from three repeats are shown on the *right. C*, after quiescence overnight in EGM-2, 0.1% FBS, hPAECs were treated with 1 ng/ml of HBMP9 or HBMP9 C73S. Samples were harvested at 1, 2, 6, and 24 h, and immunoblotting was carried out as described in *B*. Band intensity of pSmad1/5/8 blots were analyzed using ImageJ, corrected by ratios obtained from the α-tubulin blot and normalized to a wild type 1-h treatment sample. Data of the mean ± S.E. from three repeats are shown on the *right. NS*, not significant.

To investigate whether the intermolecular disulfide bond is required for BMP9 signaling activity, a HBMP9 C73S mutant was generated where Cys-73 was substituted with Ser, and it could only exist as the M-form. After quantification of the mature BMP9 in the conditioned media using the prodomain Western blot (Fig. 4*A*), a signaling assay using Smad1/5/8 phosphorylation was carried out in hPAECs. As shown in Fig. 4*B*, although slightly less active than wild type at low concentrations, C73S mutant is active in inducing Smad1/5/8 phosphorylation. However, a time course experiment revealed that at 1 ng/ml, whereas wild type BMP9 signal lasted for at least 6 h, HBMP9 C73S almost completely lost its signaling activity at 2 h (Fig. 4*C*). This indicates that although the intermolecular disulfide bond did not affect BMP9 receptor binding and the ability to signal, it could affect its stability and hence the half-life of BMP9.

##### BMP9 Is Regulated by Redox Potential

**FIGURE 5.**
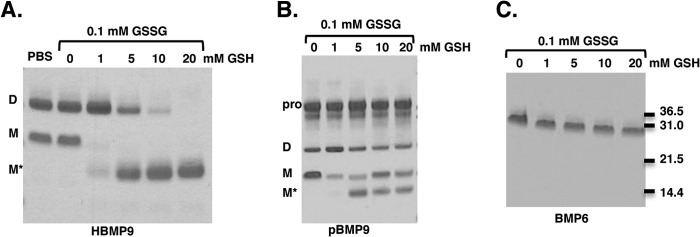
**BMP9 is regulated by redox potential.** Purified HBMP9 (*A*), pBMP9 (*B*), or BMP6 (*C*) were incubated at room temperature overnight with PBS alone or redox buffer containing 0.1 mm GSSG and 0–20 mm GSH. Proteins were then run on a non-reducing SDS-PAGE and detected by Coomassie Blue. M* is the fully reduced form of BMP9.

To investigate the stability of BMP9, we first asked whether D- and M-forms of BMP9 could be converted into each other. Incubating HBMP9 with 0.5 mm thiol-oxidizing agent diamide (41) overnight could not promote intermolecular disulfide bond formation (data not shown), indicating that the −SH groups from two Cys-73 in the non-covalently linked dimer are not in a distance readily forming a disulfide bond, consistent with our and previous observations that they are 4.8 Å apart (32). Redox buffer comprising 0.1 mm GSSG and 0 to 20 mm GSH can provide an oxidized to reduced redox range allows disulfide bond exchange and has been previously used to demonstrate a redox switch in circulating angiotensinogen. It has been shown that the angiotensinogen redox switch is reduced in plasma from healthy individuals and oxidized in plasma from preeclampsia patients (36). When HBMP9 was incubated in such redox buffer, the M-form was very sensitive to the presence of the redox buffer, readily converted into the D-form under oxidizing conditions (0.1/1 mm GSSG/GSH), and the totally reduced form (M*-form, with all three intramolecular disulfide bonds reduced) under all other conditions. The D-form BMP9 was more stable and gradually converted into M*-form under more reducing conditions (Fig. 5*A*). Because BMP9 circulates in blood as the prodomain-bound form, we investigated whether the presence of the prodomain would protect BMP9 from redox changes (Fig. 5*B*). Similar to HBMP9, in the presence of prodomain, M-form BMP9 was readily converted into the D-form and M*-form under the oxidizing and reducing buffers, respectively, although there were still some M-form left in all conditions. The D-form BMP9 could be reduced to the M-form in the reducing buffer (Fig. 5*B*, 0.1/10 mm GSSG/GSH), but there were still significant amounts of the D-form remaining even at the most reducing condition tested, supporting a role for the prodomain in stabilizing the BMP9 structure. To investigate whether this redox sensitivity was unique for BMP9, we incubated BMP6 in the same redox buffer. Purified BMP6 was a disulfide-linked dimer without the prodomain (Fig. 5*C*). Although there was a trend toward faster migration with increasing concentration of GSH, probably due to the reduction of intramolecular disulfide bonds, BMP6 remained as disulfide-linked dimer at the redox range tested and no monomeric form of BMP6 could be detected either in M- or M*-form. This indicates that the BMP6 intermolecular disulfide bond is likely to be more stable than that of BMP9 and may require the intramolecular disulfide bonds to be reduced before the intermolecular disulfide bond can be reduced. Hence, labile intermolecular disulfide bond and redox regulation is not a general feature for BMPs but may be unique to BMP9.

##### BMP9 Is Susceptible to Redox-dependent Proteolysis

**FIGURE 6.**
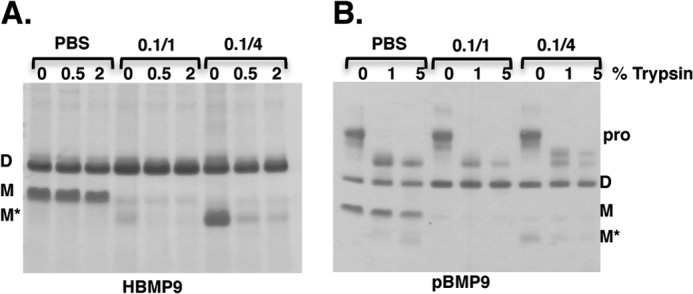
**M-form BMP9 is more susceptible to redox-dependent proteolysis.** HBMP9 (*A*) or pBMP9 (*B*) was incubated in PBS or redox buffer containing 0.1/1 mm GSSG/GSH (0.1/1) or 0.1/4 mm GSSG/GSH (0.1/4) at room temperature overnight. The following day, an aliquot of each treatment was subjected to limited trypsin digestion at 37 °C, 3 h for HBMP9 or overnight for pBMP9. Reactions were stopped by addition of SDS-loading buffer and boiling at 100 °C for 10 min. Cleavage was monitored by fractionating samples on a 12% non-reducing SDS-PAGE and Coomassie Blue staining.

We next applied limited trypsin proteolysis to probe whether there is any difference in the stability between D-, M-, and M*- forms of BMP9. HBMP9 (Fig. 6*A*) or pBMP9 (Fig. 6*B*) were incubated in PBS alone, oxidizing redox buffer (0.1/1 mm GSSG/GSH) or slightly reducing redox buffer (0.1/4 mm GSSG/GSH) overnight before limited proteolysis by trypsin. More reducing conditions containing higher concentrations of GSH were not included due to the inactivation of trypsin, probably due to the reduction of the disulfide bonds in trypsin (data not shown). As shown in Fig. 6*A*, BMP9 is unusually stable without any redox buffer and resistant to 2% trypsin digestion at 37 °C for 3 h. After overnight incubation with oxidizing redox buffer, M-form BMP9 had almost completely disappeared, mostly converted into the D-form, which was highly resistant to trypsin digestion, although a small amount of M*-form could be seen and was fully degraded even by 0.5% trypsin. Under 0.1/4 mm GSSG/GSH, the level of the D-form BMP9 had not changed compared with PBS control, but almost all the M-form BMP9 was converted into M*-form and readily cleaved by 0.5% trypsin. In the presence of the prodomain, BMP9 was slightly more resistant to trypsin. But when higher trypsin concentrations and overnight incubation were used, similar redox-dependent cleavage of the M-form could be observed (Fig. 6*B*).

##### M-form BMP9 Is Preferentially Cleaved in Serum from Control Subjects

**FIGURE 7.**
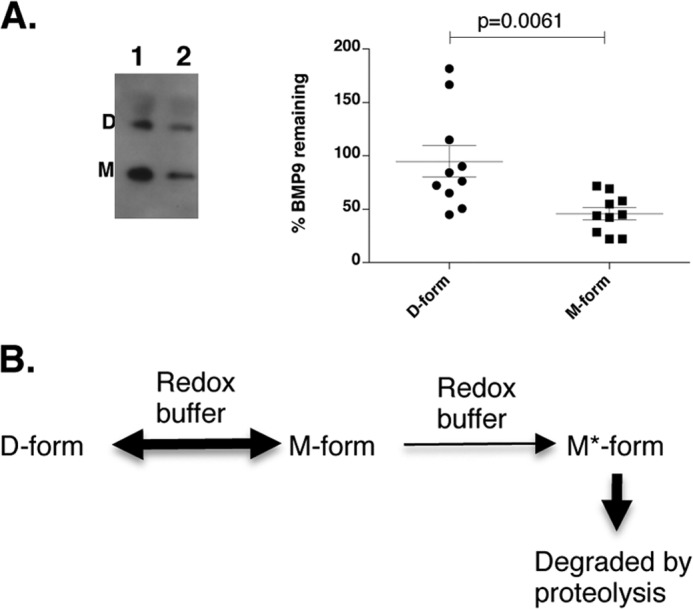
**M-form BMP9 is preferentially cleaved in serum and model of BMP9 regulation by redox-dependent proteolysis.** *A*, pBMP9 (0.3 μg) was reconstituted into 10 μl of serum (10 healthy human controls), and equal aliquots of samples were taken at 0 h (*lane 1*) and after overnight incubation at 37 °C (*lane 2*). Samples were then fractionated on a 12% SDS-PAGE under non-reducing conditions and detected by anti-BMP9 antibody (AF3209). The D- and M-forms of BMP9 at 0 h and overnight were quantified using ImageJ, and the % BMP9 remaining after overnight incubation was calculated and plotted using GraphPad Prism. Data are mean ± S.E. (*n* = 10). *B*, model of the regulation of BMP9 concentration by redox-dependent proteolysis.

We next probed whether there is evidence of redox-dependent proteolysis *in vivo* because there is GSSG/GSH redox buffer in serum (42). As the levels of circulating BMP9 are reported to be 0.07–10 ng/ml (18, 19, 27), too low to be detected by Western blot, we reconstituted pBMP9 into 10 control human serum samples and investigated whether there were any protease activity in serum that could cleave pBMP9. Aliquots were taken at 0 h and after overnight incubation at 37 °C, and the BMP9 levels were measured by Western blotting for BMP9 (AF3209, control experiments showed that this antibody can detect the D- and M-forms of BMP9 equally well, data not shown). As shown in Fig. 7, after overnight incubation at 37 °C, although there was an overall small reduction in the D-form BMP9, the amount of M-form had decreased significantly.

## DISCUSSION

TGFβ family ligands are powerful pleiotropic cytokines that function at very low concentrations. With more than 30 ligands signaling through seven type I and five type II receptors in human, there is a large degree of promiscuity in ligand-receptor recognition. Thus, their activities need to be tightly regulated both temporally and spatially. Dysregulated BMP signaling has been associated with a number of diseases characterized by bone and tissue remodeling. Whereas TGFβ is stored in a latent complex with its prodomain and activated upon binding to integrin (43), BMPs are secreted in the active forms and mostly regulated by BMP antagonists, including noggin and chordin (44). Although noggin inhibits most of the BMPs, it does not inhibit BMP9 or BMP10 (45). Among the many known BMP antagonists, only crossveinless 2 (CV2, also called BMPER) has been shown to bind and inhibit BMP9 activity (46). However, the mechanism of crossveinless 2 function is not fully understood as it has been shown that it can act as both an activator and an inhibitor of BMP signaling depending on the concentrations (47). Other factors regulating the stability and degradation of BMPs have not been well documented. Limited number of reports include the cleavage of the two sites within the BMP4 prodomain directing the intracellular trafficking and degradation of mature BMP4 (48) and megalin, a low-density lipoprotein receptor-related protein, mediating the clearance of BMP4 in the neuroepithelium (49).

BMP9 is constitutively secreted from the liver in an active form into the circulation. Mechanisms controlling its bioavailability need to be in place to ensure the optimum signaling activity and specificity. Based on our findings that BMP9 protein level can be regulated by redox-dependent proteolysis, we propose the following model as one way to control the BMP9 levels in circulation (Fig. 7*B*). BMP9 is unique among the BMP family in that stable dimers can form with or without the intermolecular disulfide bond and the equilibrium is determined by the redox potential. The two forms of BMP9 dimer show minimal differences in structure and protein-protein interactions. But the M-form is highly sensitive to the redox buffer and can be converted into the D-form under oxidizing environment or the fully reduced M*-form that is readily cleaved by proteases in the normal sera from healthy subjects. This regulation by redox-dependent cleavage may provide a natural degradation pathway in humans, in addition to the inhibition by CV2, to maintain BMP9 at low concentrations for ALK1-specific signaling.

It is difficult to provide *in vivo* evidence for this model or the presence of the M-form in nature: the circulating level of BMP9 is too low for detecting the M-form or BMP9 cleavage products, and our results would predict that the M-form would be most susceptible to degradation in the circulation. We have attempted BMP9 immunoblotting using liver samples from mice, but the multiple bands on the blots prevent the robust interpretation of the results (data not shown). However, there were several indirect lines of evidence supporting that our observations are likely to be relevant to the *in vivo* setting. First, using a pulse-chase assay, it has been shown that when pro-BMP7 was expressed and processed in CHO cells, the supernatant has the exactly same pattern of processed and partially processed BMP7 as we observed for pro-BMP9, including the monomeric forms of BMP7 (37). It was hypothesized that the close spacing of the disulfide bonds in the BMP may promote the disulfide bond exchange that resulted in the monomeric form (37). Our observation that the thiol-oxidizing agent diamide could not promote Cys-73 disulfide bond formation, but redox buffer could, also supports a role for disulfide exchange in the D- and M-form conversion. Second, in a previously published BMP9 structure in which BMP9 was generated from a special CHO cells, BMP9 was crystallized in the presence of the prodomain and existed exclusively in the M-form (32).

Apart from the many recent reports on the role of BMP9 signaling in the endothelial cell biology and cardiovascular diseases, BMP9 is also a neurotrophic factor, potently inducing and maintaining the cholinergic phenotype in the central nervous system (50). Administration of BMP9 was effective in reversing the Aβ42 amyloid plaque burden and reversing cholinergic neuron abnormalities in a mouse model of Alzheimer disease (51). BMP9 clearly shows therapeutic potential in cardiovascular diseases, neurodegenerative diseases, as well as the widely explored bone and cartilage defects. This study has provided new insights into the structure and regulation of BMP9, and these findings have broad implications for our understanding of the regulation of BMP9 *in vivo* and have provided essential information for developing BMP9 for therapeutic use.

## Footnotes

5 The abbreviations used are:

BMP: bone morphogenetic protein
ALK1: activin receptor-like kinase 1
BMPR-II: bone morphogenetic protein receptor type II
ECD: extracellular domain
pBMP9 (pHBMP9): prodomain bound BMP9 (HBMP9) complex
hPAEC: human pulmonary artery endothelial cell
PAH: pulmonary arterial hypertension
pSmad1/5/8: phosphorylated Smad1/5/8
EGM: endothelial growth medium
D-form (M-form): dimeric (monomeric) form on the non-reducing SDS-PAGE.
